# Polyarteritis Nodosa Presenting as Digital Gangrene and Breast Lesion following Exposure to Silicone Breast Implants

**DOI:** 10.1155/2015/765170

**Published:** 2015-12-30

**Authors:** Yamen Homsi, John Andrew Carlson, Samer Homsi

**Affiliations:** ^1^Division of Rheumatology, Albany Medical Center, Albany, NY 12208, USA; ^2^Division of Pathology, Albany Medical Center, Albany, NY 12208, USA; ^3^Pulmonary Department, Little Rock Diagnostic Clinic and Baptist Health Medical Center, Little Rock, AR 72205, USA

## Abstract

Polyarteritis nodosa (PAN) is a rare systemic necrotizing vasculitis of small and medium sized arteries. We report a case of a 49-year old woman who presented with PAN following exposure to silicone breast implants. Although the relationship between silicone implants and connective tissue diseases has been investigated in the literature, no prior reports were found documenting PAN after silicone mammoplasty. While the pathogenesis of idiopathic PAN is not known yet, responsiveness to immunosuppressive therapy may suggest an immunologic mechanism. More robust research is needed to understand the connection between silicone breast implants and autoimmunity.

## 1. Introduction

The relationship between silicone breast implementation and connective tissue disease has been an issue of debate for many years. In 1964, Miyoshi et al. [1] first described the term human adjuvant disease (HAD) in two patients who developed HAD after silicone mammoplasty. Polyarteritis nodosa (PAN) is a rare systemic necrotizing vasculitis of small and medium sized arteries.

To our knowledge, polyarteritis nodosa after silicone mammoplasty has not been reported in the literature to date. In this case, we report a 49-year-old female patient who developed polyarteritis nodosa following exposure to silicone breast augmentation.

## 2. Case Presentation

A 49-year-old female with a past medical history of bilateral silicone breast augmentation presented to the emergency room (ER) complaining of tingling, pain, and blue discoloration of the tips of multiple fingers for a week, along with new onset left breast black plaques. The patient reported that, for the previous two months, she had noted some redness, warmth, and swelling in inferior portion of her left breast. She received two courses of outpatient antibiotic for presumed infectious mastitis, without any improvement. In fact, the breast area had started to turn black. A few days before her presentation to the ER, she saw her primary care physician, who did a skin punch biopsy of the left breast lesion. Because of worsening finger pain, the patient decided to come to the ER.

In the hospital, a complete review of systems revealed right leg weakness, which started approximately seven days earlier, causing unsteady gait and multiple falls. The patient denied having any fever or chills. She had no left breast drainage or nipple discharges. She denied any headache, change in vision, or upper extremities weakness. She also denied any chest pain, shortness of breath, or coughing, abdominal pain, or change in bowel movement or urination. No skin rash was observed. No weight loss or night sweats were noted. The patient had no history of purpura or Raynaud's phenomenon. The patient had no arthralgia, myalgia, livedo reticularis, or weight loss. The patient is postmenopausal.

The patient has a past medical history of congenital breast asymmetry for which she underwent bilateral breast augmentation in 1991 using silicone implants. Since then, the patient underwent multiple breasts implants revisions, with the most recent revision in 2007. The patient also has a history of celiac disease and anxiety. In terms of social history, the patient denied smoking or any recreational drug use but was a former alcohol abuser.

On examination, vitals measured were temperature 36.2 c, blood pressure 136/62, heart rate 86, respiratory rate 14 per min, and oxygen saturation 99% (on room air). The patient was alert and oriented. Head, ear, eye, and nose throat were within normal limits and pupils were equal and reactive to light and accommodation. She had no neck lymphadenopathy. Heart showed a normal S1 and S2, no murmur or gallop. Lungs were clear, no crackles. Breast exam on the right was within normal: however, the left exhibited firm, tender, multiple oval black eschars just below the areola, surrounded by erythema; the largest one was about 8 cm × 4 cm at the 6 o'clock position, without discharge or fluctuance. Abdominal exam showed no tenderness, no organomegaly, and positive bowl sounds. Extremities examination showed blue discoloration of the 2, 3, 4, 5 fingers of the right hand and second and third of left hand. Nails had splinter hemorrhage. Cranial nerves were grossly intact. Speech was normal. Strength was 4/5 of right leg including straight leg raise, flexion, and extension of the knee, 5/5 left leg. Decreased left knee reflex. Sensation was intact.

Patient was started on broad-spectrum antibiotics and admitted to the hospital. Laboratory data included white blood cells of 10,300/mm, hemoglobin of 9.6 g/dL, and hematocrit of 30%. The level of C-reactive protein was 136 mg per liter (reference value, <8), and the erythrocyte sedimentation rate was 81 mm per hour (reference range 0 to 15). Laboratory testing of liver, coagulation markers, renal function, urinalysis, and urine toxicology screen were normal. Other normal or negative blood tests included complement levels, antinuclear antibody (ANA) and Anti-Neutrophil Cytoplasmic Autoantibody (ANCA), rheumatoid factor (RF), hepatitis B surface antigen, hepatitis B core antibody, hepatitis C antibody, cryoglobulin and serum, and urine protein electrophoresis. Hepatitis B surface antibody was positive. Blood cultures were performed.

In the workup for right leg weakness, the patient underwent a head CT and brain MRI, neither of which showed any acute findings, mass, or infarction. Transthoracic echocardiogram was negative for vegetation.

**Figure 1:**
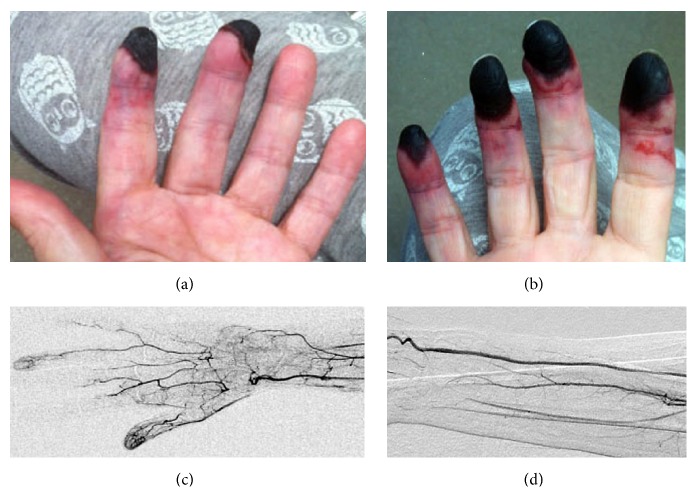
Digital gangrene. (a) and (b) show digital gangrene affecting 2nd and 3rd fingers, and the tips of the second to fifth digits of the left and right hands, respectively. (c) is an angiogram of the left hand showing occlusion of the radial artery at the wrist with lack of filling of the superficial and deep palmar arches. (d) is an angiogram of the right forearm showing occlusion of the ulnar artery proximal to the wrist.

Because of worsening digital ischemia, she was started on anticoagulation and underwent an angiogram for upper extremities. In the left arm, the left brachial artery was patent. The left radial artery was patent down to the wrist, at which point it occluded. The ulnar artery occluded just distal to its origin. The superficial and deep palmar arches were occluded (Figure 1). In the right arm, the right brachial artery was patent. The right radial artery was patent. The ulnar artery was occluded proximal to the wrist. There was partial filling of the deep palmar arch; the superficial palmar arch was occluded (Figure 1).

The differential diagnosis at this point centered on breast malignancy, infectious versus marantic endocarditis, and infectious mastitis, vacuities (giant cell arteritis or granulomatosis with polyangiitis (previously known as Wegener's granulomatosis)), and breast implant complications were also in the differential diagnosis.

Two days after admission, the pathology report of left breast punch biopsy done at her primary care office came back negative for malignancy and was significant only for superficial, neutrophilic dermatosis. During hospitalization, her ischemic fingers progressed to superficial digital gangrene (Figure 1). There was no fever spike, and blood cultures remained negative.

**Figure 2:**
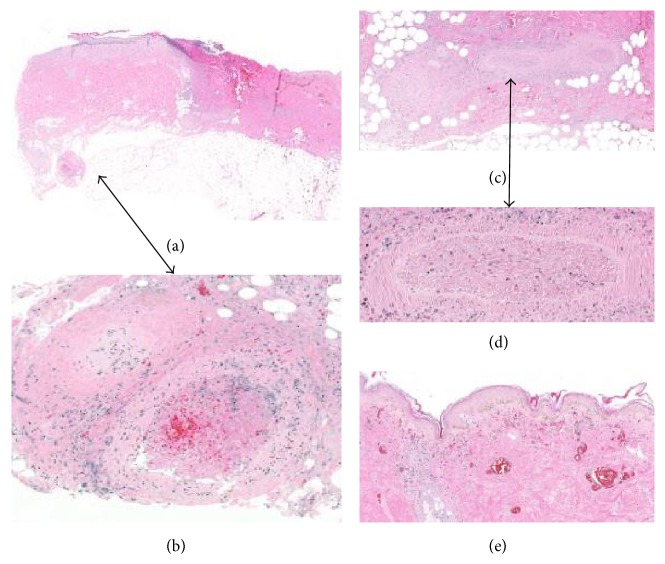
Necrotizing cutaneous polyarteritis nodosa. (a) and (b) exhibit a nodule of arteritis in the subcutis (arrows). (c) and (d) show extensive arterial damage seen as both angiodestructive inflammation and thrombotic occlusion. (e) highlights vascular congestion and extensive coagulative necrosis of epidermis and dermal adnexae. Extensive necrosis of adipocytes (see (c)) was also found indicating that arteritis caused significant ischemic damage to the surrounding tissues.

**Figure 3:**
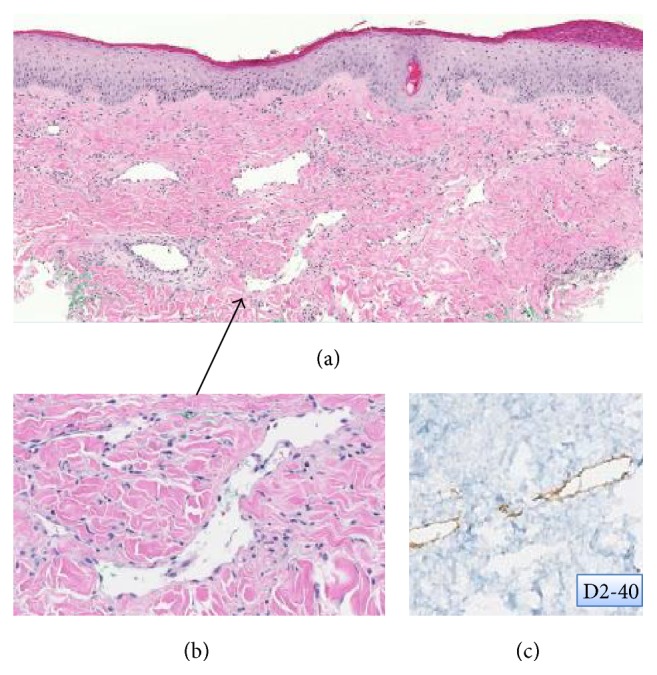
Cutaneous breast scar lymphedema. (a) shows a dermal scar with numerous dilated lymphatic vessels in a background of fibrosis. (b) highlights a markedly dilated lymphatic vessel (arrow). (c) confirms the presence of lymphangiectases by D2-40 expression (lymphatic marker).

Due to black eschar formation on the breast, the patient underwent a left breast partial mastectomy, open capsulotomy, and removal of the right implant. Histopathologic examination revealed arteritis, active and chronic consistent with polyarteritis nodosa (PAN) (Figure 2), arising in the background of scar lymphedema (Figure 3).

The patient was started on prednisone 60 mg/d and received three doses of cyclophosphamide 15 mg/kg on days 0, 30, and 60 as induction therapy and was then placed on azathioprine (2-3 mg/kg) as maintenance. She had intolerance to azathioprine; therefore she was switched to mycophenolate mofetil (1 g/bid) and maintained on it for a year. The site of the breast surgery healed completely, and she had undergone amputation of the affected digits. After one-year follow-up, patient is stable without any recurrence of her vasculitis.

## 3. Discussion

The relationship between silicone breast implants and connective tissue diseases such as systemic sclerosis (SSc), systemic lupus erythematous (SLE), and rheumatoid arthritis (RA) has been suggested in the literature. Many published reports and cases have raised concerns about the link between both.

In a large retrospective cohort study of 395,543 female health professionals, based on self-reported connective tissue disease, a total of 10830 women reported breast implants and 11800 reported connective tissue diseases between 1962 and 1991; a relative risk of 1.25 (95% CI: 1.08–1.41) for all defined connective tissue diseases was suggested [2]. However, meta-analysis published by Janowsky et al., which did not include the previous study, failed to show such a link [3].

Shoenfeld described autoimmune/inflammatory syndrome induced by adjuvants, Shoenfeld's syndrome, which included four conditions, namely, silicosis, the Gulf War syndrome (GWS), the macrophagic myofasciitis syndrome (MMF), and postvaccination phenomena. These four entities proposed the immune mediated phenomena following exposure to environmental factor including silicone (i.e., breast implant) [4].

A review article of silicone and autoimmunity by Hajdu et al. reviewed in detail the local and systemic adverse events, autoantibodies (i.e., anti-silicone antibodies) following silicone implantation. Defined autoimmune disorders such as rheumatoid arthritis and systemic sclerosis and nondefined autoimmune silicone related disorders were described too [5].

Very little is known and published about the relationship between vasculitis and silicone exposure. ANCA associated vasculitis has been suggested in setting of exposure of silicone from breast implant. Gregorini et al. [6] found that patients with ANCA positive rapidly progressive glomerulonephritis (RPGN) were 14.0 times more likely to have been exposed to silica dust than their matched control subjects (95% confidence interval, 1.7 to 113.8; *p* < 0.001). Tan et al. [7] report the second case of microscopic polyangiitis following silicone breast augmentation. Similar observation was described after implementation of ventriculoperitoneal shunt tube made of silicone [8].

PAN can present as single organ disease, for example, cutaneous polyarteritis nodosa (CPAN) described formally in 1931 by Lindberg [9], or as a multiorgan disease, or classic/systemic PAN, described in 1866 by Kussmaul [10]. Unlike ANCA associated vasculitis (e.g., granulomatosis with polyangiitis, eosinophilic granulomatous with polyangiitis, or microscopic polyangiitis), PAN is not associated with ANCA. Hepatitis B virus-associated polyarteritis nodosa (HBV-PAN) is the most typical form of PAN. In HBV-PAN, the pathogenesis is thought to be due to the deposition of circulation immune complexes in vessel wall [11]. In our case, poor lymphatic drainage, witnessed by scar lymphedema, may have allowed the deposition of immune complexes in the vessels walls and the triggering of breast arteritis.

While the pathogenesis of idiopathic PAN is not known yet, responsiveness to immunosuppressive therapy may suggest an immunologic mechanism. To our knowledge, this is the first case reporting PAN following exposure to silicone from breast implant. Additional clinical cases and scientific observation are needed to deepen the understanding of the relationship between silicone implants and vasculitis.

## References

[1] Miyoshi K., Miyaoka T., Kobayashi Y., Itakura T., Nishijo K. (1964). Hypergammaglobulinemia by prolonged adjuvanticity in man: disorders developed after augmentation mammoplasty. *The Japanese Medical Journal*.

[2] Hennekens C. H., Lee I.-M., Cook N. R. (1996). Self-reported breast implants and connective-tissue diseases in female health professionals: a retrospective cohort study. *The Journal of the American Medical Association*.

[3] Janowsky E. C., Kupper L. L., Hulka B. S. (2000). Meta-analyses of the relation between silicone breast implants and the risk of connective-tissue diseases. *The New England Journal of Medicine*.

[4] Shoenfeld Y., Agmon-Levin N. (2011). ‘ASIA’—autoimmune/inflammatory syndrome induced by adjuvants. *Journal of Autoimmunity*.

[5] Hajdu S. D., Agmon-Levin N., Shoenfeld Y. (2011). Silicone and autoimmunity. *European Journal of Clinical Investigation*.

[6] Gregorini G., Ferioli A., Donato F., Gross W. L. (1993). Association between silica exposure and necrotizing crescentic glomerulonephritis with P-ANCA and anti-MPO antibodies: a hospital-based case-control study. *ANCA-Associated Vasculitides: Immunological and Clinical Aspects*.

[7] Tan J., Spath F., Malhotra R., Hamadeh Z., Acharya A. (2014). Microscopic polyangiitis following silicone exposure from breast implantation. *Case Reports in Nephrology*.

[8] Bohgaki M., Mukai M., Notoya A., Kohno M., Takada A. (2003). Vasculitis following implantation of a ventriculoperitoneal shunt tube made of silicone. *Modern Rheumatology*.

[9] Lindberg K. (1931). Ein Beitrag zur Kenntnis der Periarteriitis nodosa. *Acta Medica Scandinavica*.

[10] Kussmaul A. (1866). *Ueber Eine Bisher Nicht Beschriebene Eigentümliche Arterienerkrankung (Periarteritis nodosa), die mit Morbus Brightii und Rapid Fortschreitender Allgemeiner Mukellähmung Einhergeht*.

[11] Guillevin L., Mahr A., Callard P. (2005). Hepatitis B virus-associated polyarteritis nodosa: clinical characteristics, outcome, and impact of treatment in 115 patients. *Medicine*.

